# THE ASSOCIATION BETWEEN FINANCIAL HARDSHIP AND MARKERS OF INFLAMMATION AMONG CANCER SURVIVORS

**DOI:** 10.1093/geroni/igad104.3764

**Published:** 2023-12-21

**Authors:** Agus Surachman, Jingxin Yao, Rose Ann DiMaria-Ghalili, Hee-Soon Juon

**Affiliations:** Dornsife School of Public Health, Drexel University, Philadelphia, Pennsylvania, United States; Drexel University, Philadelphia, Pennsylvania, United States; Drexel University, Philadelphia, Pennsylvania, United States; Thomas Jefferson University, Philadelphia, Pennsylvania, United States

## Abstract

This study examined the cross-sectional association between financial hardship and inflammation markers among cancer survivors. Furthermore, we tested if financial hardship mediated the link between education and inflammation. We used data from 304 cancer survivors (Ages = 32-90, 51% female, 81% non-Hispanic white) in the Midlife in the United States (MIDUS) wave 3 and Refresher Biomarker studies. Financial hardship was based on three domains: material (e.g., lower income to poverty line ratio), psychosocial (e.g., difficult paying bills), and behavioral (e.g., cut back on spending). We included interleukin-6 (IL-6) and c-reactive protein (CRP) as inflammation markers. Analyses were adjusted for confounders, including age, race/ethnicity, sex, marital status, body mass index (BMI), and smoking status. Analysis was conducted using the structural equation model framework. The second-order measurement model of financial hardship with three domains (material, psychosocial, and behavioral) in the first order showed a satisfactory model fit. Higher financial hardship was significantly associated with elevated CRP (Est = 0.14, SE = 0.06, p < .01) but not IL-6 (Est = -0.07, SE = 0.05, p = .13). Furthermore, financial hardship partially mediated the association between education and CRP (indirect effect: Est = -0.03, SE = 0.02, p < .05). Inflammation is a major biological pathway associated with socioeconomic disparities in long-term chronic health conditions among cancer survivors. Explicating socioeconomic conditions contributing to this disease process beyond traditional socioeconomic indicators are critical to identifying potential intervention areas to narrow the disparities.

